# Are perfectionistic strivings beneficial or detrimental to well‐being and achievement? Tests of procrastination and emotion regulation as moderators

**DOI:** 10.1111/jopy.12955

**Published:** 2024-06-17

**Authors:** Tanja Lischetzke, Gloria Grommisch, Elisabeth Prestele, Christine Altstötter‐Gleich

**Affiliations:** ^1^ Department of Psychology University of Kaiserslautern‐Landau Landau Germany

**Keywords:** perfectionistic strivings, self‐regulation, procrastination, emotion regulation, ambulatory assessment, latent variable interactions

## Abstract

**Objective:**

Researchers have called for an approach that examines the conditions under which perfectionistic strivings (PS) may be beneficial or detrimental to psychological functioning. We adopted a self‐regulation perspective and tested whether individual differences in self‐regulation (procrastination, emotion regulation) moderate PS's relationships with achievement and well‐being in an academic/work‐related achievement context.

**Method:**

A sample of 183 preservice teachers participated in a study that combined “traditional” longitudinal assessment (six performance situations over a 9‐month period) with repeated ambulatory assessment (measuring well‐being, procrastination, and emotion regulation during a total of 910 preparation phases that preceded performance situations).

**Results:**

Mean levels of achievement, well‐being, and emotion regulation were found to be stable over time, whereas procrastination decreased on average across preparation phases. Results from latent variable interaction models indicated that individual differences in intraindividual change in procrastination over time moderated the relationship between PS and well‐being (but not achievement) in the expected direction: The less individuals decreased in procrastination over time, the more negative the relationship between PS and well‐being was. Contrary to expectations, we found no evidence of a moderating effect of emotion regulation.

**Conclusion:**

The study contributes to a nuanced perspective on the adaptiveness of PS.

## 1 INTRODUCTION

Perfectionism is characterized by two higher‐order dimensions: perfectionistic strivings (PS) and perfectionistic concerns (PC; e.g., Stoeber & Otto, 2006). PS encompass the setting of and striving for exceedingly high standards for oneself and one's performance. PC refer to the overly critical evaluation of goal attainment and encompass doubts about the quality of one's performance, concerns over making mistakes, and the feeling that there is a discrepancy between one's actual achievements and one's high expectations. Although the two perfectionism dimensions are typically positively related in empirical studies, they are differentially associated with characteristics that indicate psychological (mal)adjustment. Higher PC have consistently been related to lower subjective well‐being (Lo & Abbott, 2013; Stoeber & Otto, 2006), higher psychopathology (Limburg et al., 2017), and higher “unhelpful” academic characteristics such as procrastination, academic burnout, or test anxiety (Osenk et al., 2020). For PS, empirical findings have been more mixed: Both positive and negative relationships with indicators of subjective well‐being have been identified (Lo & Abbott, 2013; Stoeber & Otto, 2006). In terms of psychopathology, meta‐analytic evidence revealed that PS show small to moderate positive bivariate correlations with symptoms of psychological disorders (Limburg et al., 2017). However, when PC are statistically controlled for, higher PS are typically associated with higher well‐being (Stoeber & Gaudreau, 2017; Stoeber & Otto, 2006) and show only small positive or even negative relationships with symptoms of psychological disorders (Limburg et al., 2017). In these partial correlation analyses, PC may have suppressed “detrimental variance” in PS, resulting in PS having more positive relationships with positive characteristics and more negative relationships with negative characteristics (for a structurally similar discussion of the functionality of attention to feelings for well‐being, see Lischetzke & Eid, 2003).

In terms of performance and achievement, higher PS have been shown to be associated with better performance on laboratory tasks (e.g., Stoeber et al., 2010) and higher academic achievement (Madigan, 2019) as well as higher scores on characteristics that support academic performance, such as academic engagement, self‐efficacy, and hardiness (Osenk et al., 2020), more effort on laboratory tasks (e.g., Stoeber et al., 2010), and higher work engagement (Stoeber & Damian, 2016). However, when individuals with high PS are confronted with life events involving achievement failure, they have been shown to be at risk for depression (Hewitt et al., 2022). Thus, the idea that PS are beneficial for psychological functioning has been controversial (e.g., Flett & Hewitt, 2006; Owens & Slade, 2008).

Personality researchers have called for more research on the adaptiveness of PS—in particular, an approach that examines the conditions under which PS may be beneficial for or detrimental to well‐being and achievement. For example, Gaudreau (2013) stated, “research on perfectionism […] has yet, for the most part, to explicate when and for whom some dimensions of perfectionism correlate with good or bad outcomes” (p. 354). In a similar vein, Gotwals et al. (2012) argued, “future research should explore the conditions and contexts that influence when the dimension [PS] will be adaptive, maladaptive, or neutral” (p. 275). However, research on *moderators* of the associations between PS and well‐being and achievement is relatively scarce. In the present research, we focused on a work‐related achievement context and aimed to test whether individual differences in self‐regulation would moderate the relationship between PS and well‐being as well as the relationship between PS and achievement.

In the remainder of the Introduction, we first distinguish between different facets of self‐regulation (behavioral vs. emotional self‐regulation) in an achievement context. Second, we delineate why individual differences in self‐regulation might play an important role in perfectionistic goal pursuit. Third, we briefly summarize findings from previous research on self‐regulation moderators of the relationships between PS and well‐being/achievement.

## 2 SELF‐REGULATION IN AN ACHIEVEMENT CONTEXT

Self‐regulation can be broadly defined as the goal‐directed process involved in attempts to modulate internal states (emotion, cognition) or behavior to achieve desired outcomes (e.g., McClelland et al., 2010; Nigg, 2017; Zeidner et al., 2000). In the present research, we focus on individual differences in behavioral and emotional self‐regulation, which are considered to play an important role in achievement situations (Duckworth et al., 2019; Harley et al., 2019; Jacobs & Gross, 2014) and have been shown to be linked to higher academic achievement (e.g., Edossa et al., 2018; Kim & Seo, 2015), higher work‐related life satisfaction (e.g., Beutel et al., 2016), and higher well‐being in achievement contexts (e.g., Beaumont et al., 2023).

Behavioral self‐regulation is conceptualized as the modification of one's actions to align them with personal goals, which are organized hierarchically (Carver & Scheier, 2000). From this perspective, a (more distal) goal to perform well in an upcoming achievement situation involves more proximal (short‐term) subgoals during the preparation phase (e.g., reading a specific book chapter, understanding, and memorizing its contents), which can be attained through actions that are planned and cyclically adapted. In achievement contexts, behavioral self‐regulatory failure occurs when individuals procrastinate—that is, when individuals “voluntarily delay an intended course of action despite expecting to be worse off for the delay” (Steel, 2007, p. 66). The ability to overcome procrastination (via specific strategies, e.g., situation selection or attention deployment) during preparation phases that lead up to performance situations can be seen as a key factor for academic achievement (Duckworth et al., 2019). Moreover, being able to efficiently move toward (daily) goals during a preparation phase without procrastinating should result in higher satisfaction with daily achievements and more pleasant affect (e.g., Hofmann et al., 2013)—that is, in higher daily well‐being.

Emotional self‐regulation (or short, emotion regulation) is a specific form of self‐regulation that refers to a process in which action is taken to shift current emotions toward desired emotions (Tamir et al., 2020). Ways to influence the intensity, the duration, and the quality of one's negative and positive emotions cover a wide range of cognitive, behavioral, and interpersonal strategies (Bryant & Veroff, 2007; Gross, 2015). In achievement contexts, negative (e.g., anxiety or shame) and positive emotions (e.g., enjoyment or pride) refer to achievement activities (e.g., studying) and their success and failure outcomes. While studying, negative emotions are assumed to impair (and positive emotions are assumed to promote) the motivation to learn, task‐related attention, and flexible learning strategies (e.g., Pekrun et al., 2023). In line with this reasoning, state anxiety has been shown to disrupt working memory processes (e.g., Vytal et al., 2013), and experiencing positive (vs. neutral) emotional states has been shown to lead individuals to set higher goals for themselves, persist longer when working on difficult tasks, and be more optimistic about task outcomes (for an overview, see Boehm & Lyubomirsky, 2008). Hence, in achievement contexts, successfully downregulating daily negative emotions and maintaining (or upregulating) daily positive emotions should make it easier for individuals to attain the short‐term (sub)goals involved in preparing for an upcoming performance situation.

### 2.1 Individual differences in self‐regulation facets as moderators of PS's links with well‐being and achievement

Striving for exceedingly high standards for one's performance can be considered a vulnerability factor that is associated with negative outcomes (e.g., depression) only under certain circumstances such as the presence of stressors (Hewitt & Flett, 1993; Smith et al., 2022). In our view, not only situational conditions but also personal conditions (i.e., an individual's standing on a trait) can be expected to moderate the associations of PS with achievement and well‐being. From a self‐regulation perspective, we would expect specific self‐regulation facets that are relevant in achievement contexts to act as a moderator variable: If individuals tend to procrastinate or if individuals have difficulties downregulating negative emotions and maintaining positive emotions during goal pursuit, high PS should raise the awareness that failure may loom and should generate stress, and hence, under these personal conditions, adverse outcomes of high PS should prevail. On the other hand, if individuals do not tend to procrastinate or if they possess high emotion regulation skills, high PS should confer benefits for well‐being during a preparation phase and for performance by motivating individuals to invest more effort and by giving them the feeling that they can fulfill their potential.

### 2.2 Previous findings on the role of self‐regulation facets in perfectionistic goal pursuit

Research on self‐regulation variables as moderators of the link between PS and psychological functioning is scarce. To our knowledge, the cross‐sectional study by Martin et al. (1996) is the only study to test an interaction between PS and trait procrastination. As outcomes, the authors studied health variables (depression and psychosomatic symptoms) and found no evidence of a PS × Procrastination interaction. To our knowledge, no studies to date have tested whether procrastination and emotion regulation moderate PS's relationship with achievement.

A few studies have examined whether coping, defined as cognitive and behavioral efforts to manage demands that are perceived as overwhelming or taxing on the individual's resources (Lazarus & Folkman, 1984), and thus a construct that has some conceptual overlap with emotion regulation,^1^ moderates the relationship between PS and well‐being indicators (e.g., Dunkley et al., 2000; Mor et al., 1995; O'Connor & O'Connor, 2003; Stoeber & Janssen, 2011; Stoeber & Otto, 2006). These studies have yielded mixed results.

A limitation of most previous studies is that the potential moderators (self‐regulation variables) and the outcome variables (indicators of well‐being or health) were measured with global trait questionnaires. Mor et al. (1995) themselves acknowledged this: “Clearly, a more suitable approach would have been to examine perfectionism and state anxiety in a naturalistic situation” (p. 222). In the present study, we sought to address this limitation by repeatedly assessing experiences and behaviors in (near) real time in naturalistic settings, as described in more detail in the next section.

## 3 THE PRESENT RESEARCH

In the present research, our aim was to examine the personal conditions under which PS may be beneficial or detrimental to achievement and well‐being. Specifically, we aimed to test whether individual differences in two facets of self‐regulation (procrastination and emotion regulation) moderate the effects of PS on achievement and well‐being in an academic/work‐related achievement context. We focused on daily procrastination and the regulation of daily negative and positive emotions during preparation phases that lead up to performance situations. To go beyond previous perfectionism research, which is still largely based on cross‐sectional studies or two‐wave longitudinal studies (Smith et al., 2022), we focused on personally relevant performance situations in real‐life settings and assessed our potential moderator variables in the preparation phases before performance situations using ambulatory assessment. Ambulatory assessment (also referred to as daily diary methods, experience sampling, or ecological momentary assessment) is a method for assessing behaviors, experiences, and environmental aspects of individuals' daily lives in naturalistic and unconstrained settings. By collecting data in real time (or near real time), ambulatory assessment helps to reduce recall bias and increase ecological validity (for an overview, see, e.g., Mehl & Conner, 2014).

Our hypotheses were formulated as follows:

Hypothesis 1a For individuals low in procrastination, PS are beneficial to achievement, whereas for individuals high in procrastination, PS are detrimental to achievement.

Hypothesis 1b For individuals high in emotion regulation, PS are beneficial to achievement, whereas for individuals low in emotion regulation, PS are detrimental to achievement.

Hypothesis 2a For individuals low in procrastination, PS are beneficial to well‐being, whereas for individuals high in procrastination, PS are detrimental to well‐being.

Hypothesis 2b For individuals high in emotion regulation, PS are beneficial to well‐being, whereas for individuals low in emotion regulation, PS are detrimental to well‐being.

To extend previous research on the role that self‐regulation facets might play in moderating PS' relationships with achievement and well‐being, for the present research, we sought to collect data during a phase in individuals' lives when self‐regulation and performing well in achievement situations are personally relevant. We focused on a specific group of individuals—preservice teachers—who repeatedly experienced stressful achievement situations (demonstration lessons) over a 9‐month period. To assess our criterion variables (achievement, well‐being) and our hypothesized moderator variables (procrastination, emotion regulation) in an ecologically valid and reliable way, we combined a “traditional” longitudinal assessment strategy (measuring achievement repeatedly across multiple demonstration lessons per person) with an ambulatory assessment strategy (measuring daily well‐being, daily procrastination, and daily emotion regulation during the preparation phases that led up to the achievement situations). We tested the formulated moderator hypotheses using a latent variable modeling framework.

## 4 MATERIALS AND METHODS

### 4.1 Study design

This study was part of a larger project focusing on self‐regulation in preservice teachers. After completing their university education, preservice teachers in Germany have to complete 1 year of practical training, which combines input and supervision in teacher education centers with practical training and supervision in schools. During the training, preservice teachers have to implement demonstration lessons that are evaluated by their supervisors. Typically, study seminars schedule six demonstration lessons per preservice teacher over the course of 9 months. Each evaluation feeds into the final grade, which is the most important job requirement.

Our study comprised an initial online survey at the beginning of the training, an online tutorial preparing participants for the repeated assessments phase, a 9‐month phase of repeated assessments, and a follow‐up online survey at the end of the training. In the 9‐month phase of repeated assessments, for each of the (approximately) six demonstration lessons, participants took a survey on goal setting for the demonstration lesson (not relevant to the present research), underwent an ambulatory assessment period before the demonstration lesson, and took a survey after the demonstration lesson. SoSci Survey software (Leiner, 2016) was used to collect the data. During the initial online survey, participants completed a demographic questionnaire and several trait measures (perfectionism and other measures not relevant to the present research). In the predemonstration lesson ambulatory assessment periods (10 days with two assessments per day), affective states and self‐regulation facets were assessed. Personalized links were sent via SMS so that participants could complete the daily surveys online on their smartphones. In the postdemonstration lesson assessments, achievement during the performance situation (and other constructs not relevant to the present research) was assessed. In the follow‐up online questionnaire, participants completed several trait measures again (which were not relevant to the present research). As part of a multimethod assessment strategy, we additionally collected ratings from preservice teachers' supervisors on preservice teachers' achievement during demonstration lessons. Approval was obtained from the Local Ethics Committee of the Department of Psychology at the University of Koblenz‐Landau (73_2016) and the Ministry of Education of Rhineland‐Palatinate, Germany.

### 4.2 Procedure

Participants were recruited in teacher education centers during information events. To be eligible, participants had to be enrolled as a preservice teacher, own a smartphone with an internet connection, and be willing to provide personal data (e.g., phone number). After signing up for the study, participants provided informed consent and completed the initial online questionnaire. Participants were remunerated €15 for each ambulatory assessment period when they completed at least 50% of the daily surveys in addition to the pre‐/postdemonstration lesson surveys. By completing more daily surveys, participants could participate in a raffle to win prizes ranging from €10 to €300. Moreover, participants could indicate whether they were interested in receiving a personal report of their scores at the end of the study.

### 4.3 Participants

One hundred ninety‐two participants signed up to participate. Three participants withdrew from the study before completing the initial online questionnaire, four completed only the initial online questionnaire, and two were excluded because they participated in only one ambulatory assessment period and completed less than 50% of the daily surveys. Hence, the data of 183 preservice teachers (84% women; age: *M* = 25.93, *SD* = 3.08, *Range*: 23–44) were analyzed.

### 4.4 Compliance and data cleaning

Teacher education centers scheduled up to eight demonstration lessons per preservice teacher. A total of 129, 142, 167, 162, 159, and 151 participants provided ambulatory assessment data (evening surveys on self‐regulation facets) for Demonstration Lessons 1 to 6, respectively. Only 22 participants provided ambulatory assessment data for Demonstration Lesson 7, and only seven participants for Demonstration Lesson 8, so we decided to use only the data provided for Demonstration Lessons 1 to 6. The final sample size consisted of 910 preparation phases nested in 183 individuals. On average, the final data set contained 4.97 preparation phases per participant (*SD* = 1.32, *Range*: 1–10). Eighty‐six participants (47%) provided data for 6 preparation phases, 51 participants (28%) for 5 preparation phases, 21 participants (11%) for 4 preparation phases, 11 participants (6%) for 3 preparation phases, 8 participants (4%) for 2 preparation phases, and 6 participants (3%) for 1 preparation phase. In total, 7810 evening surveys were recorded across the preparation phases that led up to Demonstration Lessons 1 to 6, and on average, participants completed 8.58 evening surveys per preparation phase (*SD* = 1.89, *Range*: 1–10). For 837 demonstration lessons, preservice teachers provided postdemonstration lesson data on achievement (*M* = 4.57 achievement assessments per person, *SD* = 1.42, *Range*: 1–5).

### 4.5 Supervisor reports

We collected ratings from preservice teachers' supervisors on (a) the general level of difficulty of the implemented teaching skills (so‐called “teaching quality facets”), which were not relevant to the present research and (b) preservice teachers' achievement during a demonstration lesson. After the supervisors gave informed consent and the preservice teachers gave us permission to ask their supervisors for a report, the supervisors were sent the surveys (by mail or email) before the supervisees underwent their demonstration lessons.

Sixty‐four supervisors completed surveys after a total of 252 demonstration lessons that 87 different preservice teachers had participated in. On average, each supervisor completed 3.94 surveys (*SD* = 2.67, *Range*: 1–13). For 203 of the demonstration lessons, supervisor reports of preservice teachers' achievement could be matched with corresponding self‐reports of achievement. Due to the limited information available, it was not possible to use supervisor‐rated achievement as the criterion variable in our models. Therefore, we used self‐reported achievement as the criterion variable. However, we used the matched self‐ and supervisor reports of achievement to examine self‐supervisor convergence (see Measures section).

### 4.6 Measures

#### 4.6.1 Perfectionistic strivings

PS were assessed with the seven‐item High Standards scale from the Almost Perfect Scale‐Revised (APS‐R; Slaney et al., 2001; e.g., “I set very high standards for myself”). Participants indicated agreement with the statements on a scale ranging from 1 (*not at all*) to 6 (*entirely*). We used a German version that has been applied in previous research (e.g., Zureck et al., 2015). As an indicator of reliability, we calculated omega (omega total; McNeish, 2018) using the R package MBESS (Kelley, 2017). Omega was 0.87. The APS‐R High Standards scale is a widely used instrument for capturing PS (e.g., Flett & Hewitt, 2015; Smith et al., 2019) and has demonstrated very high correlations with other measures of the higher order dimension of PS (e.g., Dunkley et al., 2012).

#### 4.6.2 Achievement

In each predemonstration lesson survey, participants were provided with the list of teaching skills (“teaching quality facets”; see the Supplemental Material for details) and were asked to check the skills that they planned to focus on during their next demonstration lesson. After each demonstration lesson, participants were asked to rate the degree of success with which they had implemented the selected teaching skills (“teaching quality facets”) on a single item (“In the demonstration lesson, I succeeded in implementing Facet X”) using a scale ranging from 1 (*not at all*) to 6 (*very much so*). A mean achievement score was calculated across all selected teaching skills. Higher scores indicated higher achievement.

#### 4.6.3 Supervisor‐rated achievement

Supervisors rated the degree of success with which preservice teachers had implemented a teaching skill (“teaching quality facet”) on a single item (“In the demonstration lesson, the implementation of facet X was…”) using a scale ranging from 1 (*not at all accomplished*) to 6 (*fully accomplished*). A mean supervisor‐rated achievement score was calculated across all teaching skills that were implemented in a demonstration lesson. Higher scores indicated higher achievement. In the subsample of matched self‐ and supervisor reports, supervisor ratings explained 36.6% of the variance in self‐reported achievement (for details on this analysis, see the Supplemental Material).

#### 4.6.4 Daily well‐being

In each morning and evening survey during a preparation phase, well‐being was measured with a short version of the pleasant‐unpleasant mood subscale of the Multidimensional Mood Questionnaire (Steyer et al., 1994), which has previously been used in ambulatory assessment studies (e.g., Lischetzke et al., 2012, 2022). Participants indicated their pleasant‐unpleasant mood on four items (unwell–well, bad–good, unsatisfied–satisfied, and unhappy–happy). The response format was a 6‐point bipolar scale with labels on each pole ranging from 1 (e.g., *bad*) to 6 (e.g., *good*). For the purpose of testing measurement invariance across preparation phases, for each item, a mean score was computed across all the surveys administered in a preparation phase (i.e., higher scores indicated higher well‐being). For our main analyses, we computed the mean across the four mood items for each preparation phase. As an estimate of reliability, we calculated two‐level omega (Geldhof et al., 2014) for preparation phases nested in participants. The within‐person omega was 0.96, and the between‐person omega was 0.98.

#### 4.6.5 Daily procrastination

In each evening survey during a preparation phase, procrastination was measured with three items from Patzelt and Opitz (2014) that we adapted to the context of preparing for a demonstration lesson. Each item tapped one of the three forms of procrastination: postponement (“I put off preparing for the demonstration lesson”), pausing (“I paused my preparation for the demonstration lesson today to do other things”), and premature termination (“I stopped preparing for the demonstration lesson earlier today to do other things than I set out to do”).^2^ We assessed participants' agreement with the statements on a binary scale (0 = *no*, 1 = *yes*). To arrive at a continuous indicator of each procrastination item during a preparation phase, we calculated the proportion of days on which participants engaged in the procrastinating behavior (using the number of days on which participants completed the procrastination items in the evening survey as the denominator). That is, higher item scores represented more procrastination during a preparation phase, and we used these item scores to test for measurement invariance. For our main analyses, we computed the mean across the three procrastination item scores for each preparation phase. As an estimate of reliability, we calculated two‐level omega (Geldhof et al., 2014) for preparation phases nested in participants. The within‐person omega was 0.70, and the between‐person omega was 0.89. Additional information on the construct validity of the daily procrastination measure can be found in the Supplemental Material.

#### 4.6.6 Daily emotion regulation

In each evening survey during a preparation phase, participants were asked to rate the intensity with which they experienced positive and negative emotions with respect to the upcoming demonstration lesson (relief, pride, confidence, anxiety, anger, and shame) during the day. Subsequently, participants were asked to indicate the extent to which they were successful at regulating these emotions during the day (e.g., Gruber et al., 2012). One item assessed the savoring of positive emotions (“How successful were you at savoring the positive emotions you experienced?”), and one item assessed the downregulation of negative emotions (“How successful were you at keeping the negative emotions you experienced at bay?”) on a scale ranging from 1 (*not at all*) to 6 (*very much*).^3^ For the purpose of testing for measurement invariance across preparation phases, for each item, a mean score across days was computed for each preparation phase (i.e., higher scores indicated more successful emotion regulation during a preparation phase). For our main analyses, we computed the mean across the two emotion regulation items for each preparation phase. As an estimate of reliability, we calculated two‐level alpha (because omega can only be calculated for at least three items; Geldhof et al., 2014) for preparation phases nested in participants. The within‐person alpha was 0.76, and the between‐person alpha was 0.83. Additional information on the construct validity of the daily emotion regulation measure can be found in the Supplemental Material.

### 4.7 Analytic strategy

To address our research question, we applied structural equation models for longitudinal data using maximum‐likelihood estimation with robust standard errors (MLR estimator) in Mplus 8.10 (Muthén & Muthén, 1998–2017). To handle missing data, we used the default setting in Mplus, which is to estimate the model under missing data theory (missing at random assumption) using all available data.^4^ Our hypotheses referred to interactions on the person level. To appropriately acknowledge measurement error, we aimed to test these person‐level moderator hypotheses using a latent variable interaction approach. Our study design corresponded to a three‐level nested data structure (days nested in preparation phases nested in persons), where one criterion variable (well‐being) was measured at the day level, and the other criterion variable (achievement) was measured at the preparation phase level. However, three‐level models cannot be estimated in combination with latent variable interactions in Mplus 8.10 (Muthén & Muthén, 1998–2017). Therefore, we decided to aggregate the variables that were measured at the day level (well‐being, procrastination, and emotion regulation) to the preparation phase level. To obtain multiple indicators of these constructs for each preparation phase for measurement invariance testing (see below), we aggregated each item separately across the days of a preparation phase. This approach also had the advantage that for the binary daily procrastination items, the resulting aggregated indicators were continuous (proportion of days on which individuals showed procrastination during a preparation phase), and hence, all models could be estimated using (robust) maximum likelihood estimation.

When longitudinal data are used in a latent variable framework, it is important to scrutinize measurement invariance over time prior to addressing substantive research questions (e.g., Widaman & Reise, 1997). Therefore, as a preparatory step, we specified latent state models to test whether strong measurement invariance (i.e., equal loadings and intercepts over time) could be established. We did so for all constructs that were measured repeatedly across the six preparation phases and for which at least two observed indicators were available on each occasion (i.e., well‐being, emotion regulation, and procrastination). Additionally, we scrutinized whether the repeatedly measured constructs showed stability in mean levels over time. The results of these preparatory analyses indicated whether, in our main analyses, a stable latent trait variable could be assumed for the repeatedly measured constructs or whether change over time would have to be modeled for a construct (by additionally specifying a latent growth factor). We present details on these preparatory analyses and their results in the Supplemental Material (and briefly summarize them in the Results section).

**FIGURE 1:**
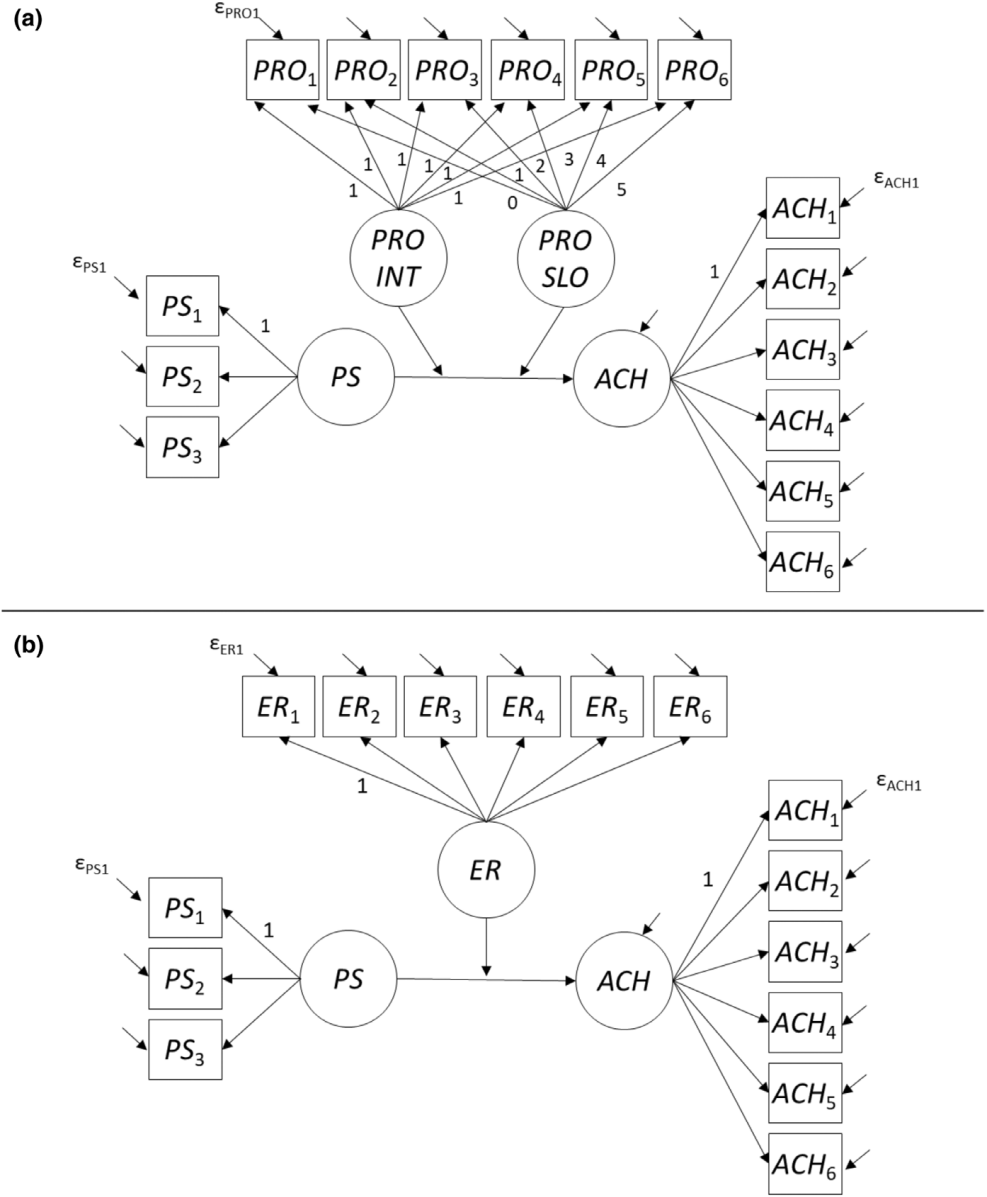
Latent variable models with achievement as the criterion variable. Panel a: Procrastination as the moderator variable. Panel b: Emotion regulation as the moderator variable. Loadings without a number were freely estimated. Intercepts are not shown in the figure. Only the first measurement error variable for each construct is labeled. ACH, latent achievement trait factor; ACH_*t*_, achievement score at demonstration lesson *t*; ER, latent emotion regulation trait factor; ER_*t*_, aggregated emotion regulation indicator for preparation phase *t*; PRO_*t*_, aggregated procrastination indicator for preparation phase *t*; PRO INT, latent procrastination intercept factor; PRO SLO, latent procrastination slope factor; PS, latent perfectionistic strivings; PS_*i*_, *i*th parcel for perfectionistic strivings; ε, measurement error.

**FIGURE 2:**
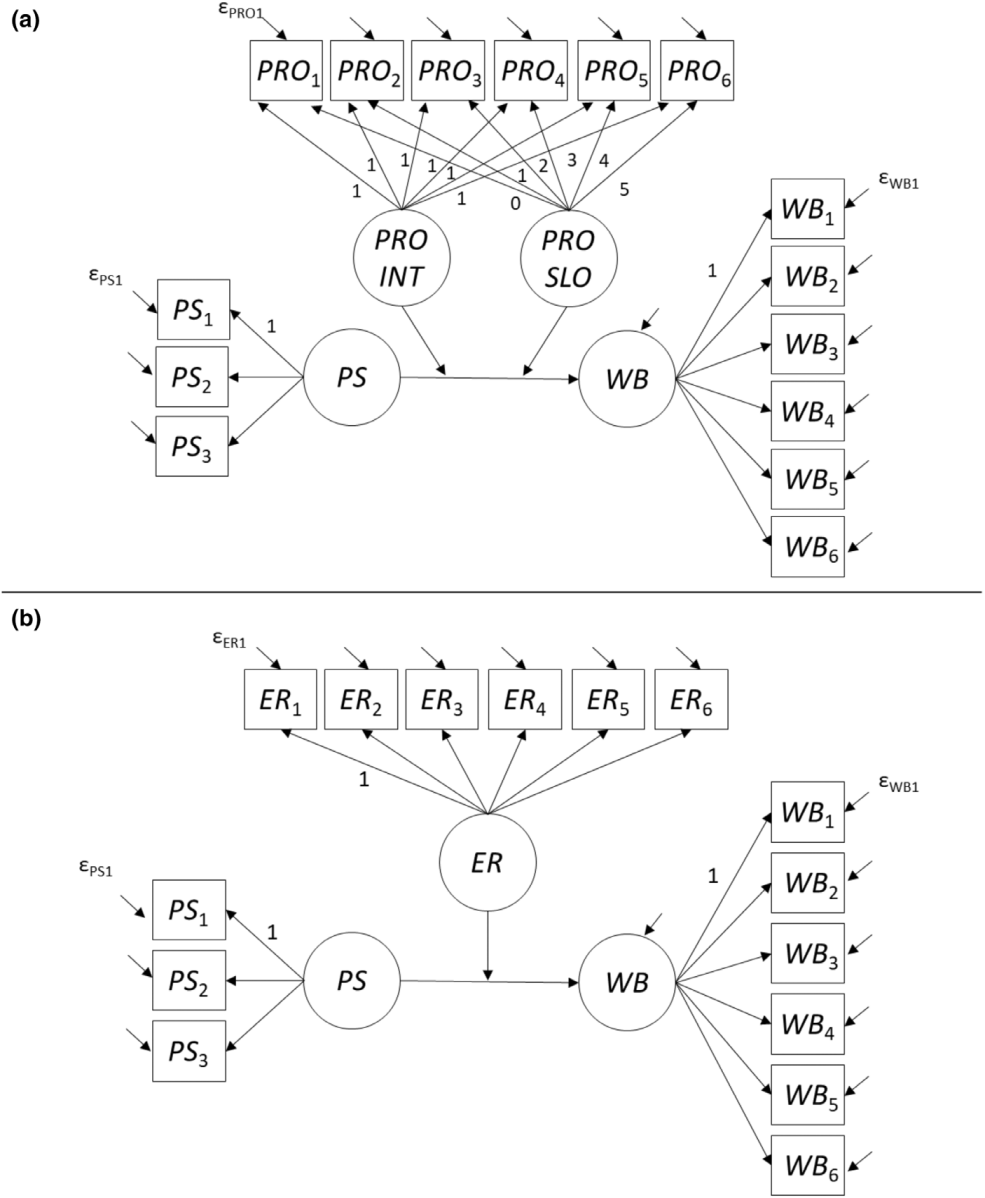
Latent variable models with well‐being as the criterion variable. Panel a: Procrastination as the moderator variable. Panel b: Emotion regulation as the moderator variable. Loadings without a number were freely estimated. Intercepts are not shown in the figure. Only the first measurement error variable for each construct is labeled. ER, latent emotion regulation trait factor; ER_*t*_, aggregated emotion regulation indicator for preparation phase *t*; PRO_*t*_, aggregated procrastination indicator for preparation phase *t*; PRO INT, latent procrastination intercept factor; PRO SLO, latent procrastination slope factor; PS, latent perfectionistic strivings; PS_*i*_, *i*th parcel for perfectionistic strivings; WB, latent well‐being trait factor; WB_*t*_, aggregated well‐being indicator for preparation phase *t*; ε, measurement error.

In our main analyses, we specified structural equation models in which the measurement model^5^ was less complex than in the models used to test for measurement invariance over time. Figure 1 illustrates the models we used to test Hypotheses 1a and 1b (with achievement as the criterion variable), and Figure 2 illustrates the models we used to test Hypotheses 2a and 2b (with well‐being as the criterion variable). PS were modeled as a latent variable (measured at the beginning of teacher training), and as observed indicators of PS, we used three item parcels, which were constructed following the item‐to‐construct balancing approach (Little et al., 2002). The criterion variables (achievement and well‐being) and the moderator variable emotion regulation were modeled as stable latent trait variables. For procrastination, preparatory analyses revealed a linear trend (decrease) across occasions (for details, see the Supplemental Material; for a summary, see the Results section). Therefore, for procrastination, we specified an intercept factor (representing individuals' initial level at the first preparation phase) and a linear growth factor (representing intraindividual change across the six preparation phases). Thus, we tested our moderator hypotheses for procrastination with two tests—one test for the latent interaction between PS and the intercept factor for procrastination and one test for the latent interaction between PS and the slope factor for procrastination. To account for multiple testing (two tests for one moderator hypothesis), we adjusted the alpha level for these tests to 0.025.

### 4.8 Sample size considerations

The present research was part of a larger project with multiple research questions. During the study planning phase, we determined the sample size based on a power analysis for an increase in *R* ^2^ in a linear multiple regression. G*Power (Faul et al., 2009) yielded a minimum sample size of 132 to detect an effect size of *ϕ* ^2^ = 0.0604 with a power of 80% and an alpha level of 5%. To account for a potential dropout rate of about 50% (across the six demonstration lessons), we originally aimed to assess 270 preservice teachers by recruiting multiple cohorts who began their training at different time points at different teacher education centers. During data collection, it turned out that recruitment took longer than planned but that the actual dropout rates were much lower than expected (see Compliance and Data Cleaning section). Therefore, we stopped recruitment when 192 participants had signed up and the maximum time period available for data collection was about to be reached.

With respect to the latent interaction effects that we set out to test in the present research (PS × Procrastination; PS × Emotion Regulation) in a structural equation modeling framework, it was not possible to conduct an a priori power analysis (Monte Carlo simulation) due to a lack of previously published latent variable analyses on self‐regulation as a moderator of the link between PS and achievement/well‐being. According to GPower (Faul et al., 2009), in a multiple regression framework (i.e., using manifest variables and hence, not accounting for measurement error), a sample size of 183 individuals would allow to achieve a power of 80% to detect an increase in *R* ^2^ (Δ*R* ^2^) of 0.035 due to the interaction term (beyond the main effects model containing two predictors and an assumed *R* ^2^ of 0.16, and assuming an alpha level of 5%). Hence, we considered our sample size large enough to test our moderator hypotheses using a latent variable framework.

### 4.9 Transparency and openness

We report how we determined our sample size, all data exclusions, and all measures used in the study. Our hypotheses were preregistered before data collection was complete and before we ran the analyses. The preregistration of hypotheses, all study materials, and the data and analysis code for this study are available online on the Open Science Framework (https://osf.io/vj5uc/). We acknowledge that certain analytic decisions (e.g., inclusion of participants based on compliance) and the analysis plan for the present research have not been preregistered.

## 5 RESULTS

### 5.1 Descriptive statistics

Table 1 presents descriptive statistics for the observed time‐varying variables at each measurement occasion. Table 2 presents descriptive statistics for PS and the (aggregated) time‐varying variables as well as within‐ and between‐person correlations between the variables.

**TABLE 1:** Means and standard deviations for the time‐varying variables at each measurement occasion.

Variable	T1		T2		T3		T4		T5		T6	
	*M*	*SD*	*M*	*SD*	*M*	*SD*	*M*	*SD*	*M*	*SD*	*M*	*SD*
ACH	4.58	0.81	4.61	0.92	4.44	0.98	4.43	1.03	4.52	1.11	4.58	0.94
WB	4.09	0.80	4.18	0.78	4.13	0.82	4.17	0.82	4.21	0.78	4.16	0.86
PRO	0.29	0.17	0.24	0.19	0.22	0.19	0.19	0.19	0.18	0.19	0.19	0.19
ER	3.91	0.85	3.97	0.75	3.91	0.85	3.99	0.89	3.99	0.84	3.98	0.90

*Note*: T1–T6 represent measurement occasions (i.e., preparation phases/demonstration lessons) 1–6, respectively. The possible range of scores was 0–1 for procrastination and 1–6 for achievement, well‐being, and emotion regulation. *N*_participants_ was 129, 142, 167, 162, 159, and 151 at T1–T6, respectively.

Abbreviations: ACH, achievement; ER, emotion regulation; PRO, procrastination; WB, well‐being.

**TABLE 2:** Descriptive statistics and correlations between the variables.

Variable	1	2	3	4	5
1. Perfectionistic strivings	—	0.13^+^	−0.09	−0.09	−0.05
2. Achievement	—	—	0.18*	−0.10	0.19*
3. Well‐being	—	0.04	—	−0.39***	0.78***
4. Procrastination	—	−0.03	0.00	—	−0.28***
5. Emotion regulation	—	0.03	0.61***	−0.01	—
*M*	4.53	4.53	4.13	0.22	3.94
*SD*_between_	0.82	0.68	0.75	0.16	0.73
*SD*_within_	—	0.72	0.38	0.12	0.47
*Range*_between_ (actual)	1.57–6.00	2.25–6.00	1.53–5.74	0–0.85	1.54–5.82
*Range*_within_ (actual)	—	1.00–6.00	1.05–5.99	0–1.00	1.14–6.00
*Range* (possible)	1–6	1–6	1–6	0–1	1–6

*Note*: *N*_participants_ = 183. Between‐person correlations are depicted above the diagonal, and within‐person correlations are depicted below the diagonal. For between‐person correlations, the time‐varying variables were aggregated (i.e., they represent participants' mean scores across the six occasions).

Abbreviations: *Range*_between_ (actual), range of observed scores at between‐person level; *Range*_within_ (actual), range of observed scores at within‐person level; *SD*_between_, between‐person standard deviation; *SD*_within_, within‐person standard deviation.

^+^ *p* < 0.10;

* *p* < 0.05;

*** *p* < 0.001.

### 5.2 Preparatory analyses

As preparatory analyses, we tested for whether strong measurement invariance over time could be established for the repeatedly measured constructs, which were measured with multiple observed indicators, and whether the constructs showed stability over time (i.e., no mean change across all preparation phases/demonstration lessons). The results of these preparatory analyses can be found in the Supplemental Material. To summarize, the results showed that strong measurement invariance could be assumed for the time‐varying constructs. Moreover, the mean levels of achievement, well‐being, and emotion regulation were stable over time. For procrastination, our preparatory analyses yielded evidence of a decline in mean levels across preparation phases. Specifically, the results of the first‐order latent growth curve model for procrastination indicated that the average intraindividual change in procrastination between two adjacent occasions (preparation phases) was estimated to be −0.020 (summing to an average total decrease in procrastination from the first to the sixth preparation phase of 0.1 on a scale of 0 to 1).^6^ Individuals differed in their estimated change in procrastination over time, as indicated by a significant variance of the procrastination slope factor. For detailed information on the distribution of the intercept and slope factors for procrastination, including significance tests for selected estimated intraindividual rates of change, readers are referred to the Supplemental Material (Tables S2 and S3). In addition, we provide information on the correlations of the procrastination intercept and slope factors with PS and the criterion variables in Table S4 in the Supplemental Material.

The results of these preparatory analyses guided our decision to model achievement, well‐being, and emotion regulation as stable latent trait variables, whereas for procrastination, we modeled both an intercept factor (representing individuals' initial levels of procrastination during the first preparation phase) and a linear slope factor (representing intraindividual change in procrastination across preparation phases) in our main analyses.

### 5.3 Main analyses

For each hypothesis, we first present results for the latent variable model that included only the main effects of the predictor variables. Second, we present results for the latent variable model that included both main and interaction effects (as depicted in Figures 1 and 2). We present these two sets of results to provide a complete picture of the predictive relationships, including information on the increment in *R* ^2^ for the models with interaction terms.

Table 3 shows the estimated parameters for the models with achievement as the criterion variable. The mean of the procrastination intercept factor (0.272 in the main effects model) can be interpreted as the average proportion of days during the first preparation phase on which individuals reported procrastination. The mean of the procrastination slope factor (−0.019) represents the average rate of change in procrastination (i.e., a decrease of 0.019 between two adjacent occasions). The main effects model (Model 1a_main) with PS and procrastination as predictor variables did not yield any significant regression coefficients. The latent variable interaction model (Model 1a_interact) showed that, unexpectedly, neither the procrastination intercept factor nor the procrastination slope factor moderated the relationship between PS and achievement. When PS and emotion regulation were analyzed as predictor variables (Model 1b_main), both higher PS and higher emotion regulation significantly predicted better achievement. Together, PS and emotion regulation explained 8.5% of the variance in achievement. However, we found no evidence of the hypothesized interaction between emotion regulation and PS in the prediction of achievement (Model 1b_interact).

**TABLE 3:** Results for the latent regression models with achievement as the criterion variable.

Main effects model								Interaction effects model							
Model Predictor	Mean	*B*	*SE*	*Std. B*	*z*	*p*	*R* ^2^	Model Predictor	Mean	*B*	*SE*	*Std. B*	*z*	*p*	*R* ^2^
*1a_main*							0.046	*1a_interact*							0.090
(Intercept)		4.604						(Intercept)		4.599					
PS	0	0.142	0.075	0.196	1.887	0.059		PS	0	0.302	0.180	0.414	1.678	0.093	
PRO‐I	0.273	−0.262	0.419	−0.069	−0.626	0.531		PRO‐I	0.272	−0.323	0.415	−0.085	−0.779	0.436	
PRO‐S	−0.019	−1.953	3.640	−0.096	−0.536	0.592		PRO‐S	−0.019	−1.550	3.434	−0.075	−0.451	0.652	
								PS × PRO‐I		−0.210	0.474	−0.041	−0.444	0.657	
								PS × PRO‐S		4.487	5.880	0.162	0.763	0.445	
*1b_main*							0.085	*1b_interact*							0.091
(Intercept)		4.569						(Intercept)		4.575					
PS	0	**0.145**	**0.072**	**0.201**	**2.024**	**0.043**		PS	0	**0.149**	**0.070**	**0.206**	**2.137**	**0.033**	
ER	0	**0.186**	**0.091**	**0.224**	**2.053**	**0.040**		ER	0	0.175	0.089	0.211	1.966	0.049	
								PS × ER		0.106	0.147	0.095	0.722	0.470	

*Note*: Bold indicates a significant regression coefficient. Mean refers to a latent factor mean. *B* denotes an unstandardized regression coefficient. *Std. B* denotes a standardized regression coefficient.

Abbreviations: ER, emotion regulation; PRO‐I, procrastination intercept factor; PRO‐S, procrastination slope factor; PS, perfectionistic strivings.

Table 4 shows the results for the models with well‐being as the criterion variable. The main effects model with PS and procrastination as the predictor variables explained 19% of the variance in well‐being (Model 2a_main). The only significant predictor in this model was the procrastination intercept factor, with higher initial procrastination predicting lower well‐being. The results for the latent variable interaction model (Model 2a_interact) revealed that the procrastination slope factor (but not the procrastination intercept factor) interacted with PS in the prediction of well‐being, and this interaction was significant at the reduced alpha level of 2.5%. To make the main effect of PS easier to interpret and to get a pure estimate of the effect size of the PS × Procrastination Slope interaction, we additionally specified a model that included only the procrastination slope factor as the moderator variable (Model 2a_interact2). In this model, the significant negative main effect of PS represents the simple slope for PS for individuals with a procrastination slope factor score of zero. That is, for individuals who did not change in procrastination across preparation phases, higher PS were significantly related to lower well‐being. The negative sign of the regression coefficient for the PS × Procrastination Slope interaction was in line with the moderating effect of procrastination we hypothesized: The higher individuals' slope coefficient for procrastination, the more negative the PS‐well‐being link was. The size of the PS × Procrastination Slope interaction was large (the interaction explained 10.6% of the variance in well‐being).^7^

**TABLE 4:** Results for the latent regression models with well‐being as the criterion variable.

Main effects model								Interaction effects model							
Model Predictor	Mean	*B*	*SE*	*Std. B*	*z*	*p*	*R* ^2^	Model Predictor	Mean	*B*	*SE*	*Std. B*	*z*	*p*	*R* ^2^
*2a_main*							0.190	*2a_interact*							0.296
(Intercept)		4.613						(Intercept)		4.676					
PS	0	−0.140	0.085	−0.146	−1.642	0.101		PS	0	−0.413	0.260	−0.432	−1.584	0.113	
PRO‐I	0.272	**−2.150**	**0.483**	**−0.430**	**−4.454**	**<0.001**		PRO‐I	0.272	**−2.134**	**0.494**	**−0.421**	**−4.321**	**<0.001**	
PRO‐S	−0.019	−4.507	3.638	−0.169	−1.239	0.215		PRO‐S	−0.019	−4.194	3.171	−0.155	−1.323	0.186	
								PS × PRO‐I		0.041	0.788	0.006	0.052	0.959	
								PS × PRO‐S		**−10.851**	**4.548**	**−0.299**	**−2.386**	**0.017**	
								*2a_interact2*							0.296
								(Intercept)		4.677					
								PS	0	**−0.402**	**0.141**	**−0.421**	**−2.856**	**0.004**	
								PRO‐I	0.272	**−2.135**	**0.490**	**−0.422**	**−4.356**	**<0.001**	
								PRO‐S	−0.019	−4.173	3.129	−0.154	−1.333	0.182	
								PS × PRO‐S		**−10.860**	**4.587**	**−0.299**	**−2.367**	**0.018**	
*2b_main*							0.736	*2b_interact*							0.737
(Intercept)		4.111						(Intercept)		4.111					
PS	0	−0.046	0.054	−0.048	−0.849	0.396		PS	0	−0.046	0.052	−0.048	−0.881	0.378	
ER	0	**0.937**	**0.072**	**0.854**	**12.976**	**<0.001**		ER	0	**0.940**	**0.074**	**0.854**	**12.721**	**<0.001**	
								PS × ER		−0.006	0.095	−0.004	−0.062	0.950	

*Note*: Bold indicates a significant regression coefficient. Mean refers to a latent factor mean. *B* denotes an unstandardized regression coefficient. *Std. B* denotes a standardized regression coefficient.

Abbreviations: PRO‐I, procrastination intercept factor; PRO‐S, procrastination slope factor; PS, perfectionistic strivings.

**FIGURE 3:**
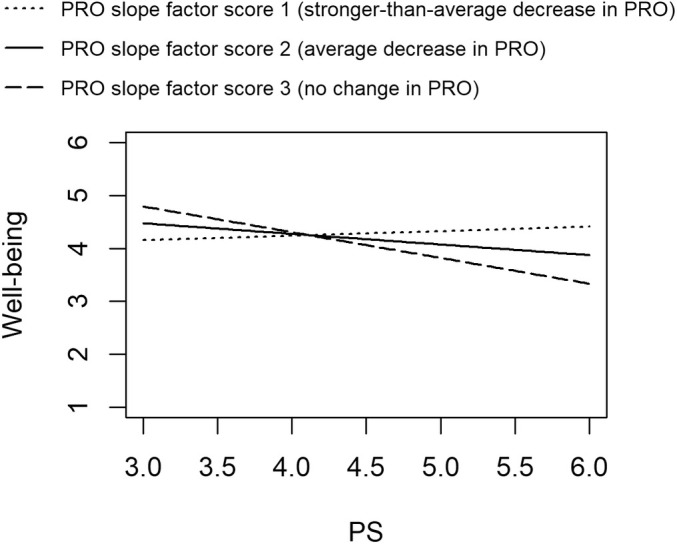
Simple slopes for the latent interaction between PS and the procrastination slope factor. PRO, procrastination; PS, perfectionistic strivings. Simple slope estimates: *b*_stronger‐than‐average decrease in PRO_ = 0.087, *SE* = 0.127, *z* = 0.684, *p* = 0.494; *b*_average decrease in PRO_ = −0.196, *SE* = 0.089, *z* = −2.210, *p* = 0.027; *b*_no change in PRO_ = −0.489, *SE* = 0.171, *z* = −2.851, *p* = 0.004.

Figure 3 illustrates the simple regression lines for the regression of well‐being on PS for three selected values of the procrastination slope factor in this model: low (*M* − 1 *SD* = −0.045, corresponding to a stronger‐than‐average decrease in procrastination over time, denoted as PRO slope factor score 1), average (*M* = −0.019, corresponding to an average decrease in procrastination over time, denoted as PRO slope factor score 2), and high (*M* + 1 *SD =* 0.008, corresponding to no change in procrastination over time, denoted as PRO slope factor score 3). The note to Figure 3 provides the significance test results for the simple slope estimates in predicting well‐being. For individuals with a stronger‐than‐average decrease in procrastination (PRO slope factor score 1), there was no significant relationship between PS and well‐being (standardized simple slope = 0.091). For individuals with an average decrease in procrastination over time (PRO slope factor score 2), the relationship between PS and well‐being was negative and significant (standardized simple slope = −0.205). For individuals who maintained their level of procrastination over time (PRO slope factor score 3), PS was even more strongly related to lower well‐being (standardized simple slope = −0.511). That is, a smaller reduction in procrastination predicted a more negative relationship between PS and well‐being. Additionally, we identified the degree of intraindividual decrease in procrastination over time at which PS were estimated to show a positive relationship with well‐being—this was the case for individuals with a change in procrastination between two adjacent occasions of (at least) −0.071 (2 *SD* below the mean procrastination slope).

When PS and emotion regulation were analyzed as predictors of well‐being, PS were unrelated to well‐being, and higher emotion regulation significantly predicted better well‐being (Model 2b_main in Table 4). The proportion of explained variance in well‐being was very high (0.736), showing that stable individual differences in emotion regulation were very strongly related to stable individual differences in well‐being across preparation phases. As can be seen from Model 2b_interact in Table 4, contrary to our expectations, emotion regulation did not interact with PS in predicting well‐being.

### 5.4 Supplemental exploratory analyses

We examined whether the results of our main analyses with procrastination as a moderator held when we controlled for individual differences in the second perfectionism dimension (PC). The results of these exploratory analyses are presented in the Supplemental Material. In summary, when achievement was analyzed as the criterion variable, the main effects model provided evidence of a suppressor effect: PS positively and PC negatively predicted achievement beyond the procrastination intercept and slope factors. The results of the interactions effects model remained unchanged when controlling for PC (i.e., no moderator effect of procrastination in the prediction of achievement). When well‐being was analyzed as the criterion variable, higher PC predicted lower well‐being, and the PS × Procrastination Slope interaction remained significant when controlling for PC.

## 6 DISCUSSION

The question of whether the perfectionism dimension PS is beneficial for psychological functioning has been controversial, and personality researchers have called for more research on the conditions under which PS may be beneficial for or detrimental to achievement and well‐being (e.g., Gaudreau, 2013; Gotwals et al., 2012). On the basis of a longitudinal study of preservice teachers who repeatedly underwent personally relevant performance situations, we tested whether two facets of self‐regulation (procrastination and emotion regulation) moderated the PS‐achievement link and the PS‐well‐being link. Going beyond previous perfectionism research, which still relies primarily on cross‐sectional or two‐wave longitudinal studies (Smith et al., 2022), we used a longitudinal design that included repeated ambulatory assessments during six preparation phases that led up to preservice teachers' performance situations (demonstration lessons) and assessed our criterion variables (achievement, well‐being) and our hypothesized moderator variables (procrastination, emotion regulation) in an ecologically valid way. By focusing on both achievement and well‐being, the present research conforms with recent recommendations (Hennecke & Bürgler, 2020) to widen criteria in studies on self‐regulation. Using a latent variable interaction approach, we found empirical support for the hypothesis that procrastination moderated the PS–well‐being link, but the other moderator hypotheses were not supported by the data.

Prior to testing our moderator hypotheses, our longitudinal design allowed us to scrutinize whether our criterion variables and our hypothesized moderator variables showed mean‐level stability across preservice teachers' repeated preparation phases and demonstration lessons. Mean levels of achievement, well‐being, and emotion regulation were stable over time. Our results revealed that, on average, procrastination decreased over time, and the average decrease from the first to the sixth preparation phase was moderate in size. We can only speculate about the reasons for this general trend of decreasing procrastination over time: One explanation is that the importance and the pressure to perform well in demonstration lessons increased for preservice teachers from the beginning to the end of the teacher training period, which (from a metacognitive self‐control perspective; see Hennecke & Bürgler, 2022) might have led to more forethought and prevention in the preparation phases (i.e., the implementation of strategies that prevent self‐control conflicts from occurring). In future process‐oriented research on self‐control, it would be interesting to explore the conditions under which individuals are successful at developing better self‐control abilities over time (e.g., Scholer et al., 2018).

Regarding the relationship between PS and preservice teachers' average well‐being across preparation phases, our results demonstrated that the bivariate correlation between PS and well‐being was small and nonsignificant. As expected, individual differences in intraindividual change in procrastination over time (but not in initial levels of procrastination or emotion regulation) moderated the relationship between PS and well‐being. The form that the interaction effect took was largely consistent with our hypothesis: For individuals who maintained their level of procrastination across preparation phases, PS demonstrated a strong negative relationship with well‐being. For individuals whose procrastination slightly decreased over time, PS demonstrated a moderate negative relationship with well‐being. A strong decrease in procrastination over time buffered the negative effect of PS on well‐being: For these individuals, PS and well‐being were unrelated. However, a positive relationship between PS and well‐being (which we had expected for individuals low in procrastination) was estimated only for individuals who showed an extremely marked decline in procrastination over time. As Smith et al. (2022) argued, “the complexities inherent in personal standards highlight the need to consider the life context” (p. 20). Our results indicate that individual differences in (intraindividual change in) procrastination during times of pressure to perform represent personal conditions under which high PS were differentially related to well‐being.

Concerning the relationship between PS and preservice teachers' achievement in demonstration lessons, our results provide some support for the notion that higher PS confer benefits for performance and achievement outcomes (e.g., Stoeber et al., 2018): Manifest PS and manifest average achievement across demonstration lessons demonstrated a small positive bivariate correlation (which was significant when using a one‐sided test), and latent PS significantly predicted higher achievement beyond emotion regulation. This result is in line with previous findings on the positive relationships between PS and academic achievement (Madigan, 2019), “helpful” academic outcomes such as academic engagement, self‐efficacy, or hardiness (Osenk et al., 2020), and better task performance (Stoeber et al., 2010). However, our hypotheses that emotion regulation and procrastination would moderate the relationship between PS and achievement were not supported by the data.

### 6.1 Limitations

In some studies, the degree of achievement during a performance situation can be operationalized in an objective way (e.g., the grade a student received in a course; Lee et al., 2003; or measured performance in a laboratory task; Stoeber et al., 2010). A limitation of the present study is that no individual grades were awarded for preservice teachers' performance in the demonstration lessons, and thus we had to rely primarily on subjective indicators of achievement. To mitigate this limitation, we additionally collected supervisor ratings of achievement (which showed high convergence with self‐ratings). However, supervisor ratings of achievement were only available for a subsample of preservice teachers and demonstration lessons. Future studies could test whether a moderating effect of emotion regulation or procrastination on the PS–achievement link can be found in an application field where degree of goal achievement can be operationalized in an objective way.

The present study has further limitations: Momentary mood (which was used as an indicator of daily well‐being) and daily emotion regulation were both measured in the evening surveys in our ambulatory assessment (momentary mood was additionally assessed in the mornings). Individuals may have used their momentary mood ratings in the evening as a basis for forming their judgments of how well they had succeeded in regulating their positive and negative emotions during the day. Such a tendency might explain why the two constructs demonstrated a very high correlation both within and between participants. Future research might try to rely less on self‐reports of emotion regulation success and well‐being by (additionally) measuring performance on emotion regulation tasks or by collecting daily reports of participants' significant others.

Finally, the advantage of studying self‐regulation, well‐being, and achievement in personally relevant performance situations in a particular population (preservice teachers) comes at the cost of having a nonrepresentative sample. Therefore, our results might not be generalizable beyond young adults in an academic setting.

### 6.2 Conclusion

Our finding of a moderating effect of individual differences in intraindividual change in procrastination over time on the relationship between PS and well‐being contributes to a nuanced perspective on the adaptiveness of PS.

## AUTHOR CONTRIBUTIONS

**Tanja Lischetzke:** Conceptualization (lead); funding acquisition (equal); methodology; data curation (equal); supervision (equal); formal analysis; writing ‐ original draft; writing ‐ review and editing (lead). **Gloria Grommisch:** Project administration; investigation; data curation (equal). **Elisabeth Prestele:** Conceptualization (supporting); data curation (equal); writing ‐ review and editing (supporting). **Christine Altstötter‐Gleich:** Conceptualization (supporting); funding acquisition (equal); supervision (equal); writing ‐ review and editing (supporting).

## FUNDING INFORMATION

This research was funded by a grant from the German Research Foundation awarded to Tanja Lischetzke (grant number LI 1827/3‐1) and Christine Altstötter‐Gleich (grant number AL 1913/2‐1). Gloria Grommisch's contribution was supported by grant GRK 2277 (Research Training Group “Statistical Modeling in Psychology”) from the German Research Foundation.

## CONFLICT OF INTEREST STATEMENT

The authors declare that they have no conflict of interest.

## ETHICS STATEMENT

Approval of the research was obtained from the Local Ethics Committee of the Department of Psychology at the University of Koblenz‐Landau (73_2016) and the Ministry of Education of Rhineland‐Palatinate, Germany.

## Supporting information


Data S1.

## Data Availability

Additional materials (preregistration of hypotheses, study materials, data, and analysis code) are available online on the Open Science Framework (https://osf.io/vj5uc/).

## ENDNOTES

^1^
Coping as a construct is linked to the occurrence of and the regulation of stress. Emotion regulation, on the other hand, may include the regulation of negative affective states such as anxiety (and thus emotion regulation strategies such as distraction used during times of stress could also be classified as coping strategies), but the emotion regulation construct is broader and also includes the regulation of positive emotions such as joy, pride, or relief (e.g., active maintenance of positive states; Bryant & Veroff, 2007).

^2^
On days on which pausing and premature termination were not applicable (because participants had not begun studying at all despite having planned to do so in the morning), only the postponement item was assessed. On days on which participants indicated in the morning survey that they did not intend to prepare for the demonstration lesson at all (and in the evening confirmed that they had not prepared for the demonstration lesson), none of the procrastination items were assessed.

^3^
Due to an error in the programming of the survey software, the response format for the downregulation of negative emotions item ranged from 1 (*not at all*) to 5 (*very much*) instead of from 1 to 6. To make the metrics of the two items equivalent, we coded the five response categories of the downregulation of negative emotions item as proportion of maximum possible (POMP) scores (i.e., 0, 0.25, 0.5, 0.75, 1) and then converted the POMP scores to a 1–6 scale (by calculating: 5 ⋅ POMP score + 1), resulting in category values of 1, 2.25, 3.5, 4.75, and 6.

^4^
Maximum likelihood estimation is a state‐of‐the‐art method for handling missing data that is preferable to deletion techniques because it results in increased statistical power and produces unbiased parameter estimates under less stringent assumptions (e.g., Enders, 2022).

^5^
For the latent criterion variables and latent moderator variables, we used one (aggregate) score for each preparation phase as observed variables. This procedure was followed to reduce the complexity (i.e., the number of parameters) of the latent variable interaction models. For the time‐varying constructs that were measured with multiple items in each preparation phase (emotion regulation, procrastination, and well‐being), we used the mean score across items as the observed indicator for each preparation phase. For achievement, a single score was available for each demonstration lesson, and hence, these scores were used as the observed indicators.

^6^
To calculate a standardized effect size for the average intraindividual change in procrastination over time, we divided the estimated change by the standard deviation of the raw procrastination scores at baseline (Feingold, 2009), which was 0.172 (see Table 1). The standardized average mean difference between two adjacent occasions was −0.116, and the standardized average total change from the first to the sixth preparation phase was −0.581.

^7^
Additional analyses were conducted to assess the impact of excluding the six participants who provided data for only one preparation phase. These analyses, based on the subset of 177 individuals who provided data for at least two preparation phases, confirmed our initial findings. In particular, the interaction between PS and the procrastination slope in predicting well‐being remained significant, supporting the robustness of our results.
