# Supplementary material for: Are perfectionistic strivings beneficial or detrimental to well‐being and achievement? Tests of procrastination and emotion regulation as moderators
Source: J Pers. 2024 Jun 17;93(3):614–32. doi: 10.1111/jopy.12955 (PMC12053826; doi:10.1111/jopy.12955)
Supplement: Supplementary file 1 — Data S1. [file JOPY-93-614-s001.pdf]

Supplemental Material

Are Perfectionistic Strivings Beneficial or Detrimental to Well-Being and Achievement? Tests of Procrastination and Emotion Regulation as Moderators

Outline of the Supplemental Material

Contents	Page
Teaching Skills (Teaching Quality Facets)	2
Convergence Between Self- and Supervisor-Rated Achievement	2
Testing for Measurement Invariance and the Equality of Means over Time	3
Estimating the Degree of Consistency for Emotion Regulation and Well-Being	5
Estimating the Degree of Change Over Time for Procrastination	5
Testing a Latent Trait Model for Achievement	7
Latent Correlations of Procrastination Intercept and Slope Factors With Perfectionistic Strivings and Criterion Variables	8
Supplemental Exploratory Analyses	8
Supplemental Analyses of the Construct Validity of the Daily Procrastination Measure	9
Supplemental Analyses of the Construct Validity of the Daily Emotion Regulation Measure and the Daily Well-Being Measure	11
References	12

Teaching Skills (Teaching Quality Facets)

The list of teaching skills ("teaching quality facets") included all the skills taught by the teacher education centers (as set by the Federal State's Ministry of Education) at the time of data collection. For preservice teachers who participated before 2017, the list consisted of the following 10 skills: Classroom management, Learning-conducive classroom climate, Motivation, Clarity and structure, Competence orientation, Dealing with heterogeneity, Support, Activation, Adequate diversity of methods, and Consolidation. In 2017, the list of teaching skills was updated by the Ministry of Education to include the following 11 skills: Classroom management, Activation and motivation, Clarity and structure, Fostering the willingness to learn, Giving support during the learning process, Giving feedback on learning processes, Continuous acquisition of skills, Fostering understanding of subject-specific contents, Acquisition of generic competencies, Performance assessment and diagnostic instruments, and Performance evaluation. Performance evaluation from the second list was selected only once by a single participant and was not considered further. On average, participants selected 3.10 (SD = 1.46) skills ("teaching quality facets") per occasion.

Convergence Between Self-Reported and Supervisor-Rated Achievement

To analyze convergence between self- and supervisor-rated achievement, we applied a mixed model to the matched data set (203 self-reports from 81 preservice teachers, 203 matched supervisor reports from 61 supervisors) using the R packages lme4 (Bates et al., 2015) and lmerTest (Kuznetsowa et al., 2017). Self-rated achievement was entered as the dependent variable, supervisor-rated achievement was entered as the independent variable, and a random intercept for preservice teachers was included (an additional crossed random intercept term for supervisors resulted in convergence problems and was therefore excluded from the model). Supervisor-rated achievement significantly predicted self-rated achievement, $b = 0.548$, $t(193.622) = 9.381$, $p < .001$, quasi-R^2 (proportional reduction in the Level 1 variance) = .366. That is, supervisor ratings explained 36.6% of the variance in self-reported achievement ratings. This is a relatively large effect size for self-other agreement in the domain of job performance (e.g., Heidemeier & Moser, 2009) and can be interpreted as an indication of convergent validity.

Testing for Measurement Invariance and the Equality of Means Over Time

We specified latent state (LS) models in Mplus 8.8 (Muthén & Muthén, 2008-2017) and tested for configural, weak, and strong invariance across occasions (i.e., preparation phases) by restricting the loadings and intercepts to equality in a stepwise procedure. For model comparisons, we applied change in the CFI (ΔCFI < 0.01; Cheung & Rensvold, 2002; Isiordia & Ferrer, 2018) as the criterion. This approach was used for any repeatedly measured constructs for which at least two observed indicators were available (emotion regulation, procrastination, well-being). Table S1 shows the model fit coefficients for the LS models with different degrees of measurement invariance. For emotion regulation and well-being, the absolute fit of the LS models was good, and for procrastination, model fit was satisfactory. The CFI did not decrease by more than .01 from one model to the next more restrictive model. We could thus assume strong measurement invariance across occasions for emotion regulation, procrastination, and well-being.

Next, we tested whether the means of the LS factors could be set equal across occasions. The model fit coefficients for the LS models with strong measurement invariance and equal state factor means over time can also be found in Table S1. For emotion regulation and well-being, the CFI did not increase by more than .01. This finding was confirmed by the results of chi-square difference tests: $\chi^2(5) = 4.371$, $p = .497$ for emotion regulation and $\chi^2(5) = 2.470$, $p = .781$ for well-being. That is, for emotion regulation and well-being, equal means over time could be assumed. For procrastination, the LS model with equal state factor means fit the data worse: The CFI decreased by .028, and the chi-square difference test was significant, $\chi^2(5) = 35.680$, $p < .001$. An inspection of the mean estimates for the LS factors revealed that, on average, procrastination decreased over time.

Table S1

Model Fit Indices for LS Models With Different Degrees of Measurement Invariance (MI)

Construct	Model	χ^2	*df*	*p*	SCF	RMSEA	90% CI (RMSEA)	SRMR	CFI
ER	Configural MI	49.522	33	.032	1.1986	0.052	[0.016, 0.081]	0.031	0.986
	Weak MI	51.331	38	.073	1.2102	0.044	[0.000, 0.072]	0.041	0.989
	Strong MI	52.608	43	.150	1.2336	0.035	[0.000, 0.064]	0.039	0.992
	**Strong MI + equal means**	**56.890**	**48**	**.178**	**1.2537**	**0.032**	**[0.000, 0.060]**	**0.047**	**0.992**
WB	Configural MI	385.197	216	<.001	1.4152	0.065	[0.055, 0.076]	0.036	0.974
	Weak MI	398.633	230	<.001	1.4685	0.063	[0.053, 0.074]	0.039	0.974
	Strong MI	418.669	245	<.001	1.5622	0.062	[0.052, 0.072]	0.039	0.973
	**Strong MI + equal means**	**421.110**	**250**	**<.001**	**1.5969**	**0.061**	**[0.051, 0.071]**	**0.039**	**0.973**
PRO	Configural MI	201.350	114	<.001	1.4499	0.065	[0.050, 0.079]	0.053	0.930
	Weak MI	213.044	124	<.001	1.4983	0.063	[0.048, 0.077]	0.058	0.928
	**Strong MI**	**237.157**	**134**	**<.001**	**1.5997**	**0.065**	**[0.051, 0.078]**	**0.061**	**0.917**
	Strong MI + equal means	276.233	139	<.001	1.6333	0.073	[0.061, 0.086]	0.092	0.889

Note. The estimator was robust maximum likelihood (MLR). Bold represents the selected model. ER = Emotion regulation. PRO = Procrastination. WB = Well-being. SCF = Scaling correction factor for MLR. RMSEA = Root mean square error of approximation. SRMR = Standardized root mean square residual. CFI = Comparative fit index.

Estimating the Degree of Consistency in Emotion Regulation and Well-Being

For emotion regulation and well-being, we extended the LS models to latent state trait (LST) models to estimate consistency (i.e., the proportion of variance in an LS factor that is due to the trait factor). Information on model fit for these LST models and the range of the estimated consistency coefficients for the six state factors can be found in Table S2. The estimates imply that for emotion regulation, between 57% and 79% of the LS variances could be attributed to between-person differences that were stable across measurement occasions. For well-being, between 62% and 82% of the LS variances could be attributed to stable differences between individuals.

Estimating the Degree of Change Over Time for Procrastination

For procrastination, we extended the LS model to a second-order latent growth curve (LGC) model. The fit of the second-order LGC model to the data was satisfactory (see Table S2). The estimated mean of the slope factor was negative (-0.024), and it was significantly different from zero, $SE = 0.004$, $z = -5.462$, $p < .001$.

For our main analyses, we planned to specify latent variable models with less complex measurement models (to reduce the complexity of the latent interaction models). Therefore, for procrastination, we additionally analyzed a first-order LGC model in which the observed indicators for each preparation phase were the mean scores across the three procrastination items. This first-order LGC model demonstrated a good fit to the data (see last row of Table S2). The estimated mean of the intercept factor was 0.273, and the estimated mean of the slope factor was -0.020 (see Table S2). The mean of the slope factor was significantly different from zero, $SE = 0.003$, $z = -6.116$, $p < .001$. In total, from the first to the sixth preparation phase, procrastination was estimated to decrease, on average, by -0.02*5 = 0.1. Note that individuals' procrastination scores can be interpreted as the average proportion of days on which procrastination behavior was reported during a preparation phase (Range: 0–1). To calculate a standardized effect size for the average intraindividual change in procrastination over time, we divided the estimated change by the standard deviation of the raw procrastination scores at baseline (Feingold, 2009), which was 0.172. The standardized average mean difference between two adjacent occasions was -.116, and the standardized average total change from the first to the sixth preparation phase was -.581.

Table S2

Model Fit Indices and Estimated Parameters for the Latent State Trait and Latent Growth Curve Models

Construct	Model	χ^2	*df*	*p*	RMSEA	90% CI (RMSEA)	SRMR	CFI	Rel	Con	*M*(Int.)	*SD*(Int.)	*M*(Slope)	*SD*(Slope)
ER	LST	72.223	57	.084	0.038	[0.000, 0.063]	0.055	0.987	.76–.92	.57–.79	—	—	—	—
WB	LST	426.387	257	<.001	0.060	[0.050, 0.070]	0.058	0.974	.94–.99	.62–.82	—	—	—	—
PRO	LGC (2nd order)	261.768	150	<.001	0.064	[0.051, 0.077]	0.073	0.910	.47–.74	—	0.332	0.169	-0.024	0.033
PRO	LGC (1st order)	25.931	16	.055	0.058	[0.000, 0.098]	0.070	0.964	—	—	0.273	0.141	-0.020	0.027

Note. ER = Emotion regulation. WB = Well-being. PRO = Procrastination. LST = Latent State Trait Model. LGC = Latent Growth Curve Model. RMSEA = Root mean square error of approximation. SRMR = Standardized root mean square residual. CFI = Comparative fit index. Rel = Reliability of observed variables, indicated as range across the different observed indicators and measurement occasions. Con = Consistency (i.e., proportion of the variance in an LS factor that is due to the trait factor), indicated as a range across the six measurement occasions. *M*(Int.) = Estimated mean of the intercept factor. *SD*(Int.) = Estimated standard deviation of the intercept factor. *M*(Slope) = Estimated mean of the slope factor. *SD*(Slope) = Estimated standard deviation of the slope factor.

Table S3

Estimated Change in Procrastination for Selected Slope Factor Scores in the First Order Latent Growth Curve Model for Procrastination

Individual slope factor score	Estimated change rate	*z*	*p*	Estimated total change	Direction of Change
Very low (*M* - 2 *SD*)	-0.073	-6.597	< .001	-0.365	Decrease
Low (*M* - 1 *SD*)	-0.046	-7.462	< .001	-0.230	Decrease
Average (*M*)	-0.020	-6.116	< .001	-0.100	Decrease
High (*M* + 1 *SD*)	0.007	1.148	.251	0.035	No change
Very high (*M* + 2 *SD*)	0.034	3.068	.002	0.170	Increase

Note. Estimated change rate = Mean difference between two adjacent occasions (preparation phases). Estimated total change = Estimated change from 1st to 6th preparation phase.

Individuals differed in the estimated change in procrastination over time, as indicated by a variance estimate of the procrastination slope factor in the first-order LGC model that was significantly greater than zero, variance estimate = 0.0007, *SE* = 0.0003, z = 2.528, p = .011. To get a better picture of the individual differences in intraindividual change in procrastination across preparation phases, we used the MODEL CONSTRAINT option in Mplus to estimate the individual rate of change (i.e., the expected mean difference in procrastination between two adjacent occasions) for selected slope factor scores. Table S3 shows the estimated intraindividual rate of change in procrastination for individuals with a “very low” (*M* – 2 *SD*), “low” (*M* – 1 *SD*), “average” (*M*), “high” (*M* + 1 *SD*), or “very high” (*M* + 2 *SD*) slope factor score.

Testing a Latent Trait Model for Achievement

For achievement, for which only one observed indicator was available at each measurement occasion (demonstration lesson), we first analyzed whether the manifest means were equal across time. A model with correlated state variables and an equality constraint on the six mean scores fit the data well, $\chi^2(5) = 6.637$, p = .249, RMSEA = 0.042, 90% CI [0.000, 0.117], SRMR = 0.032, CFI = 0.982. The results of a first-order LGC model, with an intercept factor and a linear slope factor for achievement, confirmed that there was no (linear) change over time for achievement. The fit of this LGC model was good, $\chi^2(16) = 16.607$, p = .412, RMSEA = 0.014, 90% CI [0.000, 0.071], SRMR = 0.078, CFI = 0.993. The mean of the slope factor (-0.005) was not significantly different from zero, *SE* = 0.020, z = -0.261, p = .794. Therefore, we removed the growth factor and estimated a one-factor model, which demonstrated a satisfactory fit to the data, $\chi^2(9) = 17.765$, p = .038, RMSEA = 0.073, 90% CI [0.017, 0.123], SRMR = 0.066, CFI = 0.905.

Latent Correlations of Procrastination Intercept and Slope Factors With Perfectionistic Strivings and Criterion Variables

To get information on how the latent intercept and slope factors for procrastination were correlated with PS and the two criterion variables (achievement and well-being), we specified a latent variable model with correlations among the latent variables. This model included a linear growth structure (i.e., intercept and slope factors) for procrastination, a latent factor for PS, and stable trait factors across occasions (preparation phases) for achievement and well-being. Model fit was good, $\chi^2(187) = 227.691$, $p = .023$, RMSEA = 0.034, 90% CI [0.014, 0.049], SRMR = 0.064, CFI = 0.972. The estimated latent correlations can be found in Table S4.

Table S4

Latent Correlations Between Procrastination Intercept and Slope Factors, Perfectionistic Strivings, Achievement, and Well-Being

Latent variable	PRO Intercept	PRO Slope	PS	Achievement	Well-Being
PRO Intercept	—				
PRO Slope	-.21	—			
PS	-.22*	.26**	—		
Achievement	-.09	-.05	.18^{+}	—	
Well-Being	-.36***	-.12	-.10	.21^{+}	—

Note. PRO = Procrastination. PS = Perfectionistic Strivings.
$^{+}$ $p < .10$. * $p < .05$. ** $p < .01$. *** $p < .001$.

Supplemental Exploratory Analyses

We explored whether the results of our main analyses with procrastination as a moderator held when we controlled for individual differences in the second perfectionism dimension, perfectionistic concerns (PC). PC were assessed with the 12-item Discrepancy scale from the Almost Perfect Scale-Revised (APS-R; Slaney et al., 2001). An example item is “I am hardly ever satisfied with my performance.” Participants indicated agreement with the statements on a scale ranging from 1 (*not at all*) to 6 (*entirely*). We used a German version that has been applied in previous research (e.g., Zureck et al., 2015). Omega was .95. PC was also modeled as a latent variable, and as observed indicators of PC, we used three item parcels (i.e., we used the same modeling approach as for PS).

When achievement was analyzed as the criterion variable (main effects model), higher PS positively predicted achievement (*B* = 0.239, *SE* = 0.097, standardized *B* = .328, *z* = 2.465, *p* = .014), the regression coefficient for PC was nonsignificant (*B* = -0.108, *SE* = 0.064, standardized *B* = -.223, *z* = -16961, *p* = .090), and the coefficients for the procrastination intercept and slope factors remained nonsignificant (explained variance in achievement: 8.2%). In the interaction effects model, the only significant predictor of achievement was PS (no other effects were significantly different from zero). That is, when adding PC to the model as a predictor of achievement, there was some evidence of a suppressor effect (resulting in an increased predictive power of PS), but the (nonsignificant) interaction effects remained unchanged.

When well-being was analyzed as the criterion variable, the main effects model with PS, PC, Procrastination Intercept, and Procrastination Slope as the predictor variables explained 35.7% of the variance in well-being. PC predicted lower well-being (*B* = -0.228, *SE* = 0.061, standardized *B* = -.357, *z* = -3.726, *p* < .001), and the pattern of the other predictors was similar to the results in Model 2a_main (i.e., a significant negative regression coefficient for Procrastination Intercept and nonsignificant regression coefficients for PS and Procrastination Slope). In the interaction effects model, results remained largely unchanged when PC was added as an additional predictor of well-being. That is, the regression coefficient for the procrastination intercept factor and the regression coefficient for the PS × Procrastination Slope interaction remained significant when controlling for PC.

Supplemental Analyses of the Construct Validity of the Daily Procrastination Measure

To examine the *convergent validity* of our daily procrastination measure, we analyzed the relationships between individual differences in daily procrastination and facets of conscientiousness. Conscientiousness was assessed in the initial online survey with the German version of the 12-item Conscientiousness scale from the Short Five (S5) questionnaire (Konstabel et al., 2012). Participants indicated agreement with the statements on a scale ranging from 1 (*not at all*) to 6 (*entirely*). The S5 conscientiousness items measure the conscientiousness facets of competence, order, dutifulness, achievement striving, self-discipline, and deliberation. Self-discipline (items “When I have started something, I finish it despite fatigue or other distractions. I always finish my tasks on time” and “I often postpone difficult or unpleasant activities and leave things unfinished. It is difficult for me to pull myself together and do the

things that I have to") captures dispositional behavioral self-regulation. As an indication of convergent validity, between-person differences in daily procrastination during the preparation phases should be closely related to self-discipline.

We specified a two-level confirmatory factor analysis model in Mplus with the three procrastination items as observed indicators of daily procrastination. At the person level, we added the six facets of conscientiousness as latent variables (measured by the 12 S5 items). The model fit was good, χ^2 (69) = 104.470, p = .004, CFI = 0.970, RMSEA = 0.024, SRMR (within) = 0.004, SRMR (between) = 0.046. Between-person differences in daily procrastination correlated significantly with competence (r = -.37, p = .002), order (r = -.40, p < .001), achievement striving (r = -.24, p = .021), and self-discipline (r = -.53, p < .001), but not with duty (r = -.17, p = .170) or deliberation (r = -.01, p = .911). That is, daily procrastination showed a high correlation with self-discipline, and that correlation was the highest of all the correlations. When person-level daily procrastination was regressed on the six facets of conscientiousness simultaneously, the only significant predictor was self-discipline (b = -0.139, SE = 0.067, z = -2.074, p = .038, stand. b = -.966). Taken together, this pattern of results can be taken as evidence of the construct validity of our daily procrastination measure.

In addition, to examine whether procrastination decreases across days within a preparation phase as the demonstration lesson (i.e., the performance situation) approaches, we specified a three-level structural equation model in Mplus (for days nested in preparation phases nested in persons). At the day level, latent daily procrastination (measured by the three procrastination items) was regressed on the time variable "day" (with day 1 of the preparation phase coded as 0). That is, a linear trajectory of latent daily procrastination across days was estimated at level 1. Note that the procrastination items are categorical (no/yes) at the day level, and that a three-level model for categorical variables requires Bayesian estimation in Mplus. The results showed a negative regression coefficient for day, b = -0.369, 95% Bayesian credibility interval = [-0.399; -0.337]. That is, the tendency to procrastinate decreased over the days of a preparation phase as the demonstration lesson approached.

Supplemental Analyses of the Construct Validity of the Daily Emotion Regulation Measure and the Daily Well-Being Measure

Finally, we addressed concerns about the validity of the daily emotion regulation measure that arose because of the high correlation between daily emotion regulation and daily well-being. To analyze the *factorial validity* of the emotion regulation and well-being measures, we conducted two-level confirmatory factor analyses in Mplus (for preparation phases nested in persons). The four well-being (mood) items and the two emotion regulation items were used as observed indicators. A model with two separate latent factors (emotion regulation vs. well-being) showed an acceptable fit to the data, χ^2 (16) = 125.806, $p < .001$, CFI = 0.970, RMSEA = 0.087, SRMR (within) = 0.025, SRMR (between) = 0.011. However, a model with a single latent factor did not fit the data well, χ^2 (18) = 267.684, $p < .001$, CFI = 0.932, RMSEA = 0.123, SRMR (within) = 0.068, SRMR (between) = 0.037. In addition, the information criteria values were higher for the one-factor model (AIC = 4897.680, BIC = 5042.084) than for the two-factor model (AIC = 4712.697, BIC = 4866.727), suggesting that the two-factor model should be preferred even if higher model complexity is penalized. That is, although daily emotion regulation and daily well-being were highly correlated in our study, the two constructs could be distinguished.

In addition, to examine the *convergent validity* of the daily emotion regulation measure, we analyzed its relationship to dispositional emotion regulation as assessed in the initial online survey (using a measure of dispositional emotion regulation that has been used in previous research, e.g., Lischetzke & Eid, 2003). We extended the two-level confirmatory factor analysis model with two latent factors for daily emotion regulation and daily well-being described above to include an additional latent factor for person-level dispositional emotion regulation (measured by three item parcels). We regressed the latent factor for dispositional emotion regulation on both latent daily emotion regulation and latent daily well-being at the person level. Daily emotion regulation significantly predicted dispositional emotion regulation (b = 1.037, SE = 0.355, z = 2.922, p = .003, stand. b = .840), whereas daily well-being did not predict dispositional emotion regulation beyond daily emotion regulation (b = -0.309, SE = 0.263, z = -1.175, p = .240, stand. b = -.314). That is, between-person differences in daily emotion regulation and dispositional emotion regulation converged strongly, which can be interpreted as an indication of construct validity.

References

Bates, D., Mächler, M., Bolker, B., & Walker, S. (2015). Fitting linear mixed-effects models using lme4. *Journal of Statistical Software*, *67*(1). https://doi.org/10.18637/jss.v067.i01

Cheung, G. W., & Rensvold, R. B. (2002). Evaluating Goodness-of-Fit Indexes for Testing Measurement Invariance. *Structural Equation Modeling: A Multidisciplinary Journal*, *9*(2), 233–255. https://doi.org/10.1207/S15328007SEM0902_5

Feingold, A. (2009). Effect sizes for growth-modeling analysis for controlled clinical trials in the same metric as for classical analysis. *Psychological Methods*, *14*(1), 43–53. https://doi.org/10.1037/a0014699

Heidemeier, H., & Moser, K. (2009). Self-other agreement in job performance ratings: A meta-analytic test of a process model. *The Journal of Applied Psychology*, *94*(2), 353–370. https://doi.org/10.1037/0021-9010.94.2.353

Isiordia, M., & Ferrer, E. (2018). Curve of Factors Model: A Latent Growth Modeling Approach for Educational Research. *Educational and Psychological Measurement*, *78*(2), 203–231. https://doi.org/10.1177/0013164416677143

Konstabel, K, Lönnqvist, J.-E., Walkowitz, G., Konstabel, K, & Verkasalo, M. (2012). The 'Short Five' (S5): Measuring personality traits using comprehensive single items. *European Journal of Personality*, *26*(1), 13–29. https://doi.org/10.1002/per.813

Kuznetsova, A., Brockhoff, P. B., & Christensen, R. H. B. (2017). lmerTest package: Tests in linear mixed effects models. *Journal of Statistical Software*, *82*(13). https://doi.org/10.18637/jss.v082.i13

Lischetzke, T., & Eid, M. (2003). Is attention to feelings beneficial or detrimental to affective well-being? Mood regulation as a moderator variable. *Emotion*, *3*(4), 361–377. https://doi.org/10.1037/1528-3542.3.4.361

Muthén, L. K., & Muthén, B. O. (1998–2017). *Mplus user's guide (8th ed.)*. Muthén & Muthén.

Slaney, R. B., Rice, K. G., Mobley, M., Trippi, J., & Ashby, J. S. (2001). The Revised Almost Perfect Scale. *Measurement and Evaluation in Counseling and Development*, *34*(3), 130–145. https://doi.org/10.1080/07481756.2002.12069030

Zureck, E., Altstötter-Gleich, C., Gerstenberg, F. X., & Schmitt, M. (2015). Perfectionism in the Transactional Stress Model. *Personality and Individual Differences*, *83*, 18–23. https://doi.org/10.1016/j.paid.2015.03.029
